# Adaptive evolution of a hyperthermophilic archaeon pinpoints a formate transporter as a critical factor for the growth enhancement on formate

**DOI:** 10.1038/s41598-017-05424-8

**Published:** 2017-07-21

**Authors:** Hae-Chang Jung, Seong Hyuk Lee, Sung-Mok Lee, Young Jun An, Jung-Hyun Lee, Hyun Sook Lee, Sung Gyun Kang

**Affiliations:** 1 Korea Institute of Ocean Science and Technology, Ansan, Republic of Korea; 2 Department of Marine Biotechnology, Korea University of Science and Technology, Daejeon, Republic of Korea

## Abstract

Previously, we reported that the hyperthermophilic archaeon *Thermococcus onnurineus* NA1 could grow on formate and produce H_2_. Formate conversion to hydrogen was mediated by a formate-hydrogen lyase complex and was indeed a part of chemiosmotic coupling to ATP generation. In this study, we employed an adaptation approach to enhance the cell growth on formate and investigated molecular changes. As serial transfer continued on formate-containing medium at the serum vial, cell growth, H_2_ production and formate consumption increased remarkably. The 156 times transferred-strain, WTF-156T, was demonstrated to enhance H_2_ production using formate in a bioreactor. The whole-genome sequencing of the WTF-156T strain revealed eleven mutations. While no mutation was found among the genes encoding formate hydrogen lyase, a point mutation (G154A) was identified in a formate transporter (TON_1573). The TON_1573 (A52T) mutation, when introduced into the parent strain, conferred increase in formate consumption and H_2_ production. Another adaptive passage, carried out by culturing repeatedly in a bioreactor, resulted in a strain, which has a mutation in TON_1573 (C155A) causing amino acid change, A52E. These results implicate that substitution of A52 residue of a formate transporter might be a critical factor to ensure the increase in formate uptake and cell growth.

## Introduction

H_2_ energy has drawn attention as an alternative energy source^1, 2^. Currently, the annual production of H_2_ is approximately 0.1 Gtons, of which 98% comes from the reforming of fossil fuels^3^: 40% of H_2_ is produced from natural gas, 30% is produced from heavy oil and naphtha, 18% is produced from coal, 4% is produced from electrolysis and approximately 1% is produced from biomass^4^. Due to the advantage of environmentally friendliness and cost-effectiveness compared with conventional chemical methods, biological H_2_ production has been extensively studied over several decades^5, 6^.

Formate can be produced efficiently from various inexpensive resources or as an end product of microbial activity, and a number of studies on formate-dependent H_2_ production have been carried^7–9^. A variety of microbes with formate hydrogen lyase (FHL) have been identified in phylogenetically diverse groups of archaea and bacteria^10–12^.

Oxidation of formate to CO_2_ and H_2_ under anoxic conditions is an endergonic process under standard condition (HCOO^−^ + H_2_O → HCO_3_^−^ + H_2_, Δ*G*° = +1.3 kJ/mol). In anaerobic syntrophic formate oxidation, the reaction is made thermodynamically possible by removal of the end product H_2_ using a methanogenic or sulfate-reducing partner^13–16^. No pure culture has ever been shown to grow on formate with hydrogen production. However, we demonstrated that *T. onnurineus* NA1 isolated from a deep-sea hydrothermal vent can grow on formate and produce H_2_^17–19^.

The genome of *T. onnurineus* NA1 has three copies of gene clusters encoding formate dehydrogenase, including the *fdh1-mfh1-mnh1* (TON_0266–0282), *fdh2-mfh2-mnh2* (TON_1563–1580) and *fdh3-sulfI* cluster (TON_0534–0540). Among those gene clusters, the *fdh2-mfh2-mnh2* gene cluster was shown to be solely essential for formate-driven growth^17, 19^: Fdh2 module oxidizes formate and Mfh2 module transfers electrons to protons, thereby generating a proton gradient across the membrane. The proton gradient is used by Mnh2 module to produce a secondary sodium ion gradient that drives ATP synthesis, catalyzed by a Na^+^-ATP synthase. A gene encoding formate transporter (TON_1573), which presumably plays a role in importing formate into the cytoplasm^17^, is located between *mfh2* gene cluster and *mnh2* gene cluster. Although the formate transporter has not been largely characterized in the order *Thermococcales*, it is predicted to belong to the formate/nitrite transporter (FNT) family (transporter classification 2.A.44)^20^.

In the present study, we employed adaptive laboratory evolution to enhance the cell growth of *T. onnurineus* NA1 on formate. Adaptive laboratory evolution allows the selection of desirable phenotypes in a laboratory environment against an applied stress and can be a powerful way to develop beneficial phenotypic characteristics of microbial strains^21^. During adaptation, genetic variations occur all over the chromosome, and beneficial mutations can improve the ability to handle the stress^22^. Previously, it has been demonstrated that the serial transfer of *T. onnurineus* NA1 under the CO condition greatly improved growth and CO tolerance^23^, due to mutations including a mutation at a putative DNA-binding protein (TON_1525 T55I).

To obtain an integrative picture of physiological and molecular changes during adaptation, growth profiles were monitored and the whole-genome sequence of the adapted strain was determined. The contribution of each mutation to the cell growth was investigated and H_2_ producing ability of the mutant was compared with that of the parent strain.

## Results

### Physiological changes of *T. onnurineus* NA1 during serial transfers on formate

**Figure 1:**
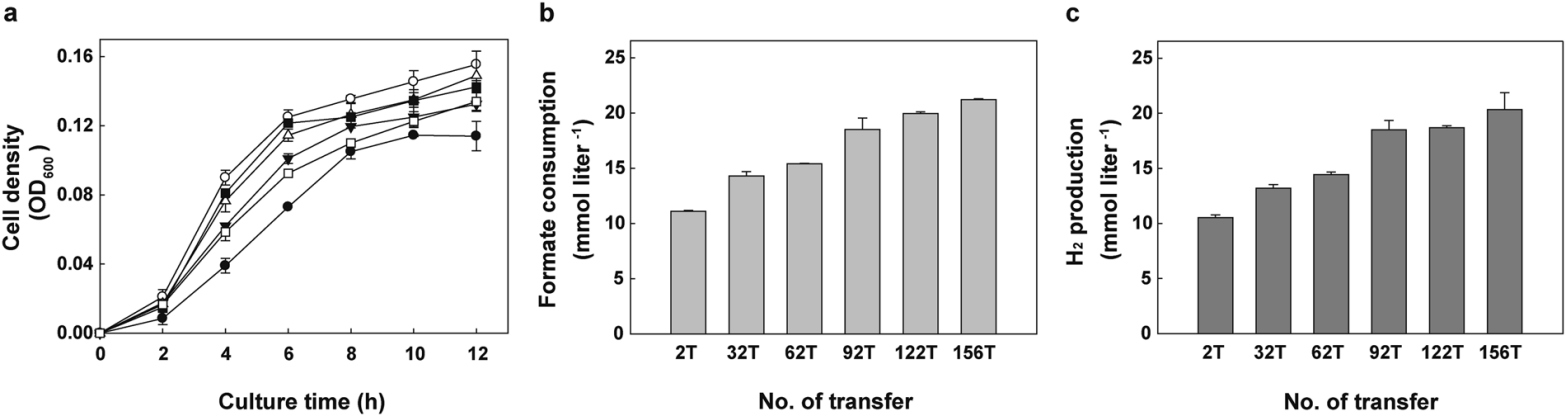
Physiological changes of *T. onnurineus* NA1 through serial transfers into fresh MM1 medium containing 147 mM sodium formate. After 2 (closed circle), 32 (open square), 62 (closed inverted triangle), 92 (open triangle), 122 (closed square) and 156 (open circle) transfers, the cell density (expressed as optical density at 600 nm) (**a**) was determined at the indicated time points. Formate consumption rates (**b**) and H_2_ production rates (**c**) were determined during the exponential phase. All experiments were conducted independently in duplicate.

For adaptive laboratory evolution of *T. onnurineus* NA1 on formate, *T. onnurineus* NA1 was inoculated into a medium containing formate as a whole energy source in a serum vial and cultured to stationary phase. Then, 2% of the culture was inoculated into the same, fresh medium and the serial transfer was repeated more than 150 times. Through these serial transfers, changes in cell growth, H_2_ production and formate consumption were monitored (Fig. 1). As the serial transfer continued, optical cell density, H_2_ production rate and formate consumption rate of *T. onnurineus* NA1 gradually increased. After 156 transfers, the adapted strain, designated WTF-156T, showed 1.71-, 1.93- and 1.91-fold higher cell density, H_2_ production rate and formate consumption rate, respectively, than the parent strain.

### Kinetic analysis of formate consumption and H_2_ production

**Figure 2:**
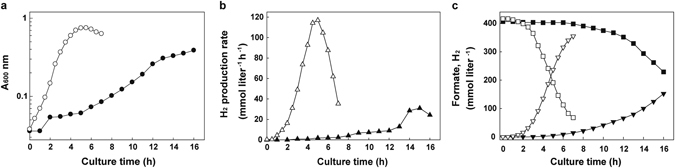
Time profiles of physiological changes in the parent and WTF-156T strains. Cell density (log *A*_*600 nm*_) (**a**) and H_2_ production rate (**b**) in the parent (closed symbol) and WTF-156T (open symbol) strains. (**c**), Changes of formate (square) and hydrogen concentrations (inverted triangle) in the parent (closed symbol) and WTF-156T (open symbol) strains during the batch culture on 400 mM sodium formate. The pH was adjusted to 6.1–6.2 using 2 N HCl containing 3.5% NaCl as a pH-adjusting agent.

Even though the adapted strain exhibited enhanced cell growth and hydrogen production on formate-containing medium, it was difficult to characterize the physiological changes quantitatively in a serum vial. The pH in the culture medium rapidly increased and the cells did not grow exponentially, with a final pH of approximately 8 at stationary phase. Therefore, the kinetic properties of WTF-156T were investigated in a pH-controlled bioreactor operated at pH 6.2 in comparison with those of the parent strain. Notably, WTF-156T exhibited a shorter lag time in the bioreactor culture. The WTF-156T strain reached 0.7–0.8 optical density (OD_600_) after 5 hours while the parent strain reached an OD_600_ of 0.39 at 16 h culture (Fig. 2a). Furthermore, the H_2_ production rate of the WTF-156T strain was higher than that of the parent strain, with maximum rates of 117 mmol liter^−1^ h^−1^ for the WTF-156T strain and 31 mmol liter^−1^ h^−1^ for the parent strain, respectively (Fig. 2b). While the parent strain consumed only 2% formate after 7 hours, WTF-156T consumed 84% during the same period of time (Fig. 2c). Formate consumption was well balanced with H_2_ production, and the molar ratios of H_2_ production per formate consumption during the batch culture remained constant at about 1:1 in both strains. The WTF-156T strain showed 1.9- and 3.8-fold higher maximum biomass yield and H_2_ production rate, respectively, than the parent strain (Table 1).

**Table 1:** Kinetic parameters of the parent and WTF-156T strains.

Kinetic parameter	Parent strain	WTF-156T strain	Fold difference
μ_max_ (h^−1^)	0.3	1.1	3.72
*r*_max_ (mmol liter^−1^ h^−1^)	31.7	109.0	3.44
Biomass productivity (g liter^−1^ h^−1^)^a^	0.026	0.101	3.89
*q*_max_ (mmol g^−1^ h^−1^)	198.2	345.7	1.74
H_2_ productivity (mmol liter^−1^ h^−1^)^b^	9.5	52.3	5.49

Kinetic parameters were calculated with data from the graphs in Fig. 2. μ_max_, maximum specific growth rate; *r*_max_, maximum H_2_ production rate; *q*_max_, maximum specific H_2_ production rate.

^a^Biomass productivity was determined by dividing total yield by time difference from 11 to 13 h for the parent strain and from 2 to 4 h for WTF-156T strain.

^b^H_2_ Productivity was determined by dividing the total yield by time.

### Genome-wide mutation analysis

To understand the cause of the physiological changes, genetic variations in the genome of WTF-156T were analysed by genome sequencing using PacBio Single Molecule Real-Time (SMRT) sequencing technology. Eleven single-base substitutions could be identified either at the coding (9 sites) or intergenic regions (2 sites) in comparison with that of the parent strain. Throughout the entire genome of WTF-156T, we found 2 insertions, 2 deletions and 7 substitutions (Supplementary Fig. 1). The base substitution occurred at genes encoding aromatic amino acid permease (TON_0820), 3-phosphoshikimate-1-carboxyvinyltransferase (TON_1138), signal peptidase (TON_1555), F_420_-reducing hydrogenase β subunit (TON_1561), formate transporter (TON_1573), hypothetical proteins (TON_0618, TON_1084, TON_1641, TON_RS08535) and noncoding regions between amino-acid transporter and biotin-protein ligase (TON_0901 -TON_0902) and between a hypothetical protein and peptide transporter (TON_1668 -TON_1669) (Table 2). To determine the time of mutation for each mutation during the adaption period, we determined the distribution of each mutation in the 2^nd^, 62^nd^ and 156^th^ transferred strains. Out of 11 mutations found in the genome of WTF-156T, 6 mutations were found in the 62^nd^ transferred strain, while the other 5 mutations were detected only in the 156^th^ transferred strain (Supplementary Fig. 2)

**Table 2:** Mutations identified in the genome of WTF 156 T strain.

Locus_tag	Location^a^	Mutational change	Product description
TON_0820	G473A	G158D	Aromatic amino acid permease
TON_1138	G993A	G331G	3-Phosphoshikimate 1-carboxyvinyltransferase
TON_1555	C485T	P162L	Signal peptidase
TON_1573	G154A	A52T	Formate transporter
TON_1641	C255T	D85D	Hypothetical protein
TON_RS08535	G257A	G86E	Hypothetical protein
TON_0618	T946 deletion	Frame shift	Hypothetical protein
TON_1084	C608 insertion	Frame shift	Hypothetical protein
TON_1561	G510 insertion	Frame shift	F_420_-reducing hydrogenase β subunit
TON_0901 -0902	A deletion at 832564^b^		between amino acid transporter and biotin-protein ligase
TON_1668 - 1669	C to A at 1532991^b^		between hypothetical protein and peptide transporter

^a^All mutations were confirmed by PCR verification and Sanger sequencing.

^b^The number indicates the genomic location.

### Effect of each mutation on the phenotypic changes of the adapted strain

**Figure 3:**
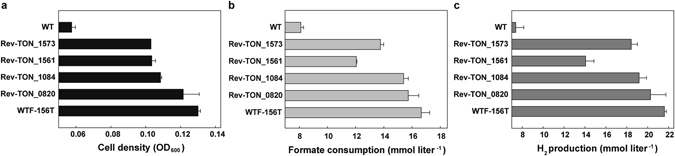
The effect of each mutation was determined by restoring each mutation in the WTF-156T strain. Cell growth (**a**), formate consumption (**b**) and H_2_ production (**c**) in the revertants were analysed in comparison with those of the parent and WTF-156T strains at the late exponential phase (after 6 h incubation). Error bars indicate the standard deviation from three independent experiments.

To evaluate the contribution of each mutation to the phenotypic changes, genes encoding aromatic amino acid permease (TON_0820), hypothetical protein (TON_1084), F_420_-reducing hydrogenase β subunit (TON_1561) and formate transporter (TON_1573) were selected before embarking on time-consuming empirical analysis. As each mutation of WTF-156T was restored to the sequence of the parent strain in the WTF-156T strain, the growth rate of all the revertants decreased (Fig. 3). In particular, Rev-TON_1561 and Rev-TON_1573 showed significant decrease in cell density and H_2_ production in comparison with the WTF-156T strain.

**Figure 4:**
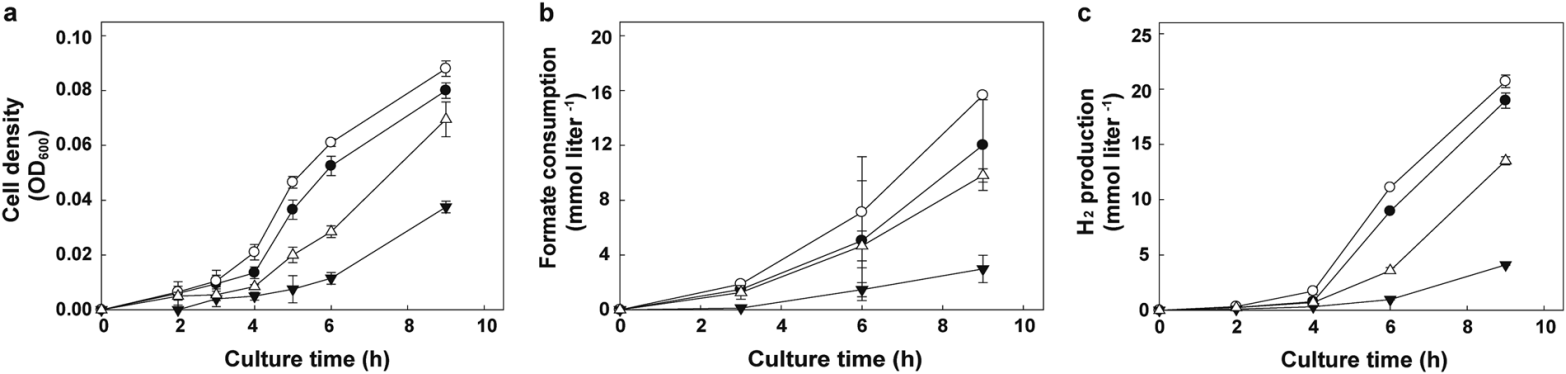
The effect of mutation at TON_1573 or TON_1561. Changes of cell density (optical density at 600 nm) (**a**), formate consumption (**b**) and H_2_ production (**c**) were determined in the parent (closed circle), TON_1573 (A52T) (open circle), TON_1561 (G510 insertion) (open triangle) and TON_1573 deleted (closed inverted triangle) strains during the batch culture. Error bars indicate the standard deviations of independent duplicate experiments.

To address whether the change in TON_1561 (G510 insertion creating a frame shift mutation) or TON_1573 (G to A mutation at nucleotide 154 creating A52T mutation) contributed to the enhanced growth, we introduced each mutation into the parent strain. The resulting mutant with the alteration at TON_1573 (A52T) displayed enhanced growth, H_2_ production and formate consumption during the batch culture whereas the TON_1561 (G510 insertion) mutant showed decrease in cell density, H_2_ production and formate consumption compared to those of the parent strain (Fig. 4). In a pH-controlled bioreactor, the mutant with A52T substitution at TON_1573 showed 1.62- and 1.27-fold increase in maximum biomass yield and H_2_ production rate, respectively, than the parent strain (Supplementary Fig. 3 and Supplementary Table 1). It seems that the mutation at TON_1573 could play a critical role in increasing H_2_ production from formate in the WTF-156T strain. The knockout mutant deficient in TON_1573 exhibited a significant decrease in the growth, formate consumption and H_2_ production.

**Figure 5:**
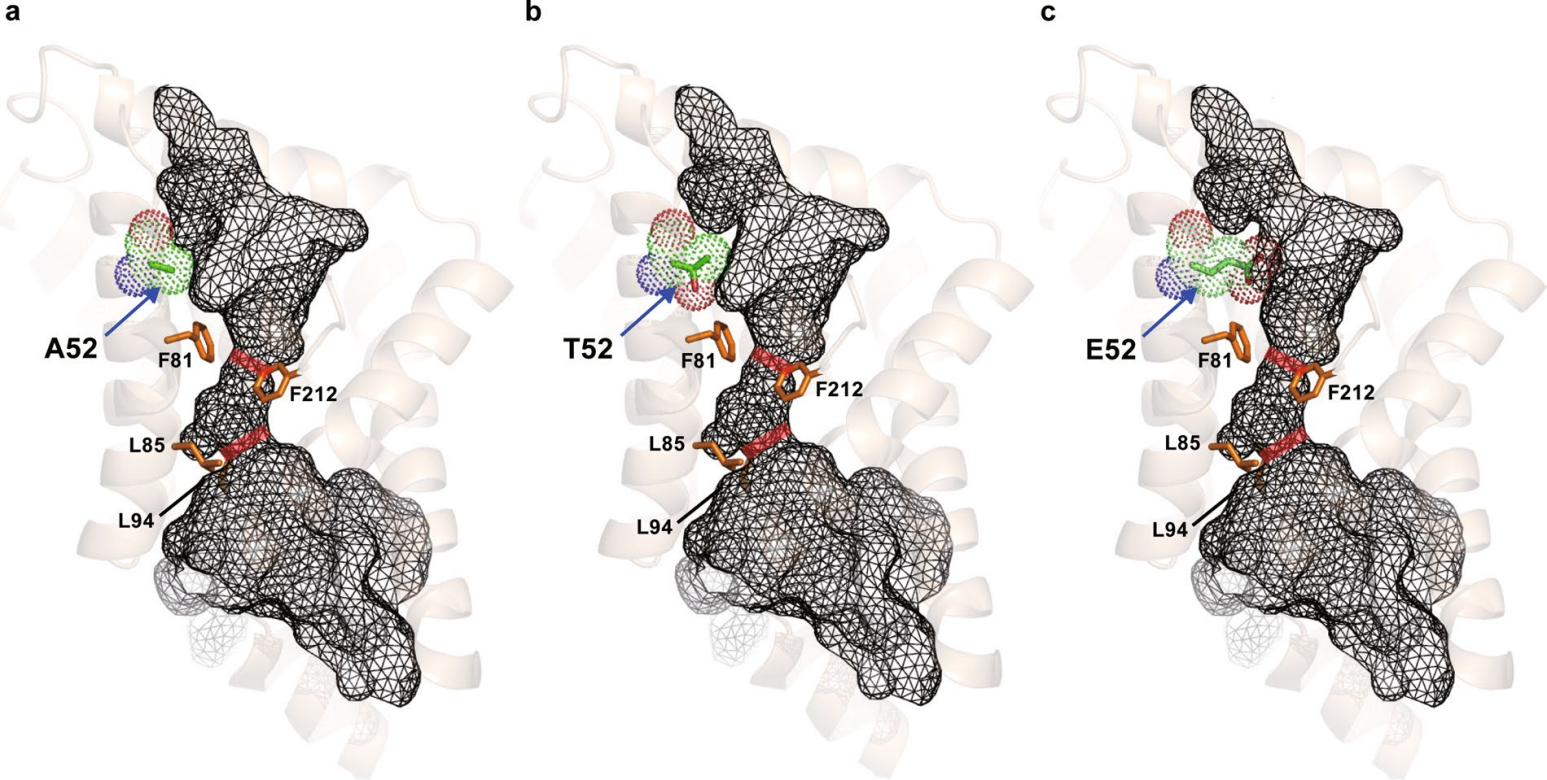
3D model structure of the formate transporter (TON_1573) in the parent strain (**a**) and A52T mutant (**b**) and A52E mutant (**c**) strains. The two predicted constriction sites in the closed central pore are highlighted in red. The F81/F212 and L85/L94 residues were predicted to contribute to the constriction sites (Supplementary Figure 4), are highlighted in orange.

TON_1573 showed homology to the formate transporter FocA, belonging to the FNT family, of the bacterial strains and thus its structure was predicted using the structure of FocA (PDB ID: 3KLY) as a template (Fig. 5). The mutated 52^nd^ residue was predicted to be part of a hydrophobic patch in the axial channel, facing internally towards the central pore. The change from alanine to threonine in the residue could slightly affect hydropathicity in the patch (Fig. 5b).

**Figure 6:**
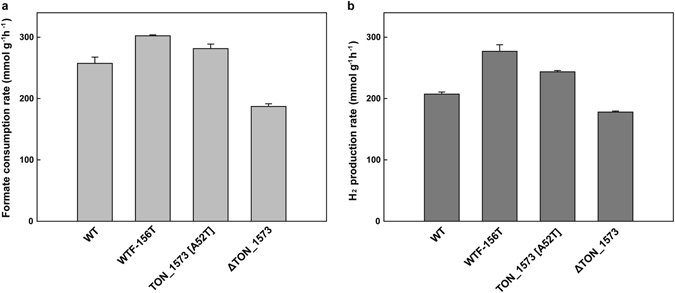
The effect of alteration at formate transporter (TON_1573) on formate uptake rate using resting cell suspensions. Formate consumption rate (**a**) and H_2_ production rate (**b**) of TON_1573 (A52T) mutant and TON_1573 deletion mutant were compared among the parent and WTF-156T strains using resting cell assay. Error bars indicate the standard deviations of independent duplicate experiments.

Apart from the experiment to investigate the effect of an amino acid residue change (A52T) in TON_1573 during the batch culture, the effect of the mutation on formate consumption was directly measured using cells suspended in a formate-containing medium as well. Cell suspensions of the parent strain and the mutant were incubated with formate, and the amount of residual formate was measured during the incubation. After incubating at 80 °C for 5 min, the WTF-156T strain showed 17.4% higher specific formate uptake rate (302.4 mmol/g/h) than the parent strain (257.6 mmol/g/h) (Fig. 6). The mutant at TON_1573 (A52T) in the parent background showed 9.3% higher specific formate consumption rate (281.6 mmol/g/h) than the parent strain. On the other hand, the deletion of TON_1573 significantly decreased formate consumption rate (187.2 mmol/g/h) and hydrogen production rate. Taken together, the A52T mutation in TON_1573 appears to be a beneficial mutation to promote cell growth by increasing formate consumption.

### Adaptation in a bioreactor

Previously, we reported the kinetic analysis of repeated batch culture of *T. onnurineus* NA1^24^, where we did not consider the possibility that the strain might be modified genetically. However, we realized that cells have been serially transferred in bioreactors through anaerobically harvesting at the end of each batch culture and inoculation into fresh medium, at least more than 10 times. Therefore, we speculated that the serial transfer in bioreactors might also cause genetic changes in the parent strain. In fact, the cell stock after the serial transfer in bioreactors showed enhanced growth and H_2_ production than the parent strain (Supplementary Table 2). Based on the assumption that genomic changes might occur in cells during the time period, the genome sequence of cells was determined using PacBio Single Molecule Real-Time (SMRT) sequencing technology. The mutations are listed in Supplementary Table 3. Interestingly, a point mutation (C155A) in TON_1573 could be identified along with several mutations. Even though C155A mutation in TON_1573 is not the same mutation as that of the previous adaptation at the serum vial (G154A), it also results in the change of the same amino acid residue, alanine to glutamate (A52E) in this case (Fig. 5c). These two independent adaptive experiments point out that the enhanced growth and formate utilization displayed in the adaptive cells was caused by a mutation at the same amino acid residue (52^nd^) of TON_1573, either A52T or A52E.

## Discussion

*T. onnurineus* NA1 has been reported to utilize C1 substrates such as CO and formate as an energy source in addition to carbohydrates^17, 25, 26^. Previously, the strain has been adapted on CO by serial transfer on CO-containing medium, accompanying a genomic change with beneficial mutations^23^. In this study, we investigated molecular changes associated with cell growth on formate as an energy source. Through more than 150 serial transfers on formate-containing medium, we observed a significant increase in formate consumption and H_2_ production. While the CO-adapted strain showed a 6.7- and 2.2-fold higher H_2_ production rate and specific H_2_ production rate, respectively, than the parent strain, the formate-adapted strain showed 3.4- and 1.7-fold increases from the corresponding values^23^. The performance in each case is, however, incommensurable at this moment due to at least the technical difference to supply substrate; CO was continuously fed at a flow rate of 400 ml min^−1^ and formate was supplemented only once at the starting point. Continuous feeding of formic acid can improve the H_2_ production rate.

During the adaptation, a total of eleven mutations were observed over 2,000 generations; the mutation rate is calculated to be approximately 0.55 per 100 generations. This mutation rate is quite similar to the estimated rate from the adaptation process on CO (0.5 mutations per 100 generations). Based on the data of these cases, the mutation rate of *T. onnurineus* NA1 seems remarkably low in comparison to those of *E. coli* and *S. cerevisiae* with 1.06 and 1.39 mutations per 100 generations, respectively^27^.

Among mutations found in WTF-156T, the mutation in formate transporter was verified to contribute to enhancing growth and hydrogen production of the parent strain. However, the mutation in F_420_ -reducing hydrogenase β subunit did not have a beneficial effect on the parent strain. The gene encoding a F_420_-reducing hydrogenase homolog in *T. onnurineus* NA1 is composed of three genes, *frhAGB*. The *frhAGB* gene shows homology to the genes encoding F_420_-reducing hydrogenase which plays a role in the methanogenesis of methanogens, but in non-methanogens including *T. onnurineus* NA1, the function of the gene product has not been clearly identified yet. The β subunit of F_420_-reducing hydrogenase from methanogens has been known to bind FAD (flavin adenine dinucleotide) and coenzyme F_420_ [*N*-(*N*-_L_-lactyl-γ-_L_-glutamyl)-_L_-glutamic acid phosphodiester of 7,8-didemethyl-8-hydroxy-5-deazariboflavin 5′-phosphate]^28^. However, the F_420_-binding motif is not well conserved in the β subunit of *T. onnurineus* NA1^29^. After a role of the β subunit of the hydrogenase complex is unveiled in *T. onnurineus* NA1, contribution of the mutation at TON_1561 during the adaptive evolution can be reevaluated in the future. As for the mutation in formate transporter, the molecular basis might be provided to address how to increase formate uptake. Based on the structural modelling, the change at 52^nd^ residue seemed to allow a subtle fit for the formate-transporting pore to favor formate transporter contributed to substrate binding through an additional hydrogen bond with formate moving through the channel. The multiple alignment of formate transporter displays that the corresponding residues to the 52^nd^ residue of TON_1573 were largely hydrophobic amino acids, such as alanine, leucine, valine and tyrosine, except serine in a formate/nitrite transporter family protein from *Clostridium butyricum* (Supplementary Fig. 4). We assume that the change of these hydrophobic residues to threonine or glutamate would enhance formate uptake from this study.

As shown in Supplementary Fig. 3 and Supplementary Table 1, the mutation at TON_1573 (A52T) was estimated to represent about 50% of the increase in formate utilization observed in the 156 T strain. So, we can’t rule out the possibility that mutational alterations in other genes might also influence on the phenotypes of the WTF-156T strain. For example, the genes encoding aromatic amino acid permease (TON_0820) and 3-phosphoshikimate-1-carboxyvinyltransferase (TON_1138) may be involved in amino acid metabolism, thus mutations on them enabling the effective utilization of yeast extract. Further studies are required to confirm the possible correlation.

Given that the mutations in formate transporter increased formate uptake and thus enhanced cell growth and H_2_ production, the capability to uptake formate can be further manipulated; by driving the expression of the gene by a strong promoter or introducing additional copies of the gene in the chromosome or episomal vector. Recently, several reports have been published on the overexpression of transporter to enhance substrate uptake and product formation. The overexpression of a hexose transporter could result in an increase of ethanol productivities probably by increasing glucose uptake in *Saccharomyces cerevisiae* ^30, 31^. In *Halomonas elongate*, overexpressed sugar transporter improved xylose consumption and the productivity of ectoine could be improved^32^. The overexpression of a galactitol transporter, which contributes to the ATP-independent xylose uptake, also increased poly(lactate-co-3-hydroxybutyrate) yields^33^.

In this study, we developed a strain which has better performance in utilizing formate and producing H_2_ through adaptive evolution process. The strain can be useful in other biotechnology applications as well as biohydrogen production, such as microbial cell factories to produce hyperthermophilic enzymes or metabolites, due to its short culture time and high biomass productivity.

## Methods

### Strain, medium, and culture condition

*T. onnurineus* NA1 (KCTC 10859), isolated from a deep-sea hydrothermal vent area^34^, was routinely cultured in modified medium 1 (MM1) at pH 6.5^17, 35^. The MM1 medium was prepared by autoclaving and being kept in an anaerobic chamber (Coy Laboratory Products, Grass Lake, MI, USA) filled with an anoxic gas mixture (N_2_/H_2_/CO_2_, 90:5:5). For the adaptive laboratory evolution study, the strain was cultured in a serum vial with the MM1 medium supplemented with 1 g·L^−1^ of yeast extract and 147 mM sodium formate at 80 °C and every 15 h, 2% of each culture was transferred to a fresh medium. To prepare cell suspensions, the strains were cultured in a 2-L Scott-Duran glass bottle containing 1 L of the MM1 medium with 1 g·L^−1^ yeast extract and 147 mM sodium formate at 80 °C for 12 h. For the pH-stat bioreactor culture, the strain was cultured on the MM1 medium with 4 g·L^−1^ of yeast extract and 400 mM sodium formate with pH controlled at 6.2 by adding 2 N HCl in 3.5% NaCl. The anaerobic culture was conducted by flushing argon gas into the bioreactor for at least 30 min before inoculation in a 3-L bioreactor (Fermentec, Cheongwon, Korea) with a working volume of 1.5 L. The culture was performed at 80 °C and agitation speed of 300 rpm.

### Analytical methods

Cell growth was measured using the optical density at 600 nm (OD_600_) with a BioPhotometer plus a UV-Visible spectrophotometer (Eppendorf, Hamburg, Germany). Biomass concentration was determined by the correlation of dry cell weight (DCW) with OD_600_ as described in a previous report^18^. The concentration of formate was measured using high-performance liquid chromatography (HPLC) equipped with a UV detector and an RSpak KC-811 column (Shodex, Tokyo, Japan) at a flow rate of 1.0 ml min^−1^ with a mobile phase of 0.1% (vol/vol) H_3_PO_4_. The concentration of H_2_ in the headspace was measured by sampling the headspace gas (100 μl) using gas-tight syringes using an YL6100GC gas chromatograph (GC) (YL Instrument Co., Anyang, Republic of Korea) equipped with a Molsieve 5A column (Supelco, Bellefonte, PA, USA), a Porapak N column (Supelco), a thermal conductivity detector and a flame ionization detector. Then, the production rate of hydrogen (mmol per liter of medium) was calculated. Argon was used as a carrier gas at a flow rate of 30 ml min^−1^. The total volume of outlet gas in a bioreactor was measured using a wet gas meter (Shinagawa, Tokyo, Japan)

### Genome sequencing

Genomic DNA was extracted from cultures of the WTF-156T strain without single-colony isolation. Whole-genome sequencing of the strain was carried out using PacBio Single Molecule Real-Time (SMRT) sequencing (Pacific Biosciences, Menlo Park, CA, USA) with a 10-kb insert library approximately 100X coverage^36^. Assembly and consensus polishing were performed using SMRTpipe HGAP and SMRTpipe Quiver, respectively^37^. The sequence was compared with that of the parent strain and variants were identified using SAMtools v0.1.18. All the mutations were verified by polymerase chain reaction (PCR) and Sanger sequencing. All the primers for PCR confirmation are listed in Supplementary Table 4.

### Construction of mutants

All the mutants were made by applying the gene disruption system^38^. Briefly, we designed primer pairs for single base-pair substitutions and mutated genes by site-directed mutagenesis. Each mutated gene and flanking regions of target gene were ligated by one-step sequence- and ligation-independent cloning (SLIC)^39^, and the subsequent mutants were generated through homologous recombination using an unmarked in-frame deletion method and a modified gene disruption system^38, 40^. Cells were transformed and incubated in the presence of 10 µM simvastatin as a selection marker. All the mutants were isolated by single colony isolation. All the primers used for introduction of mutation, gene disruption and verification of constructs are given in Supplementary Table 4.

### Cell suspension experiment

Cells were harvested at the end of the culture by centrifugation at 8,000 × *g* at 25 °C for 20 min. Cells were washed to remove residual formate with a buffer containing 20 mM imidazole-HCl (pH 7.5), 600 mM NaCl, 30 mM MgCl_2_ and 10 mM KCl. Cell suspensions of an OD_600_ of 0.5 in the MM1 medium were preincubated for 30 min at 80 °C in a rubber-sealed glass vial. The reaction was initiated by the addition of 50 mM sodium formate. Gas and culture samples were taken at time intervals for GC and HPLC analyses.

### Protein structure modeling

Three-dimensional (3D) structure of TON_1573 protein was modeled on the basis of the crystal structure of FocA (PDB ID: 3KLY) selected by its lowest e-value. The homology model was generated by SWISS-MODEL server (http://swissmodel.expasy.org/) for automated comparative modeling of 3D protein structure and analysed using the PyMOL molecular graphics system version 0.99 (http://www.pymol.org/).

### Data availability statement

All data generated or analysed during this study are included in this published article and Supplementary files.

## Electronic supplementary material

Supplementary Figures and Tables
