# Supplementary material for: Adaptive evolution of a hyperthermophilic archaeon pinpoints a formate transporter as a critical factor for the growth enhancement on formate
Source: Sci Rep. 2017 Jul 21;7:6124. doi: 10.1038/s41598-017-05424-8 (PMC5522443; doi:10.1038/s41598-017-05424-8)
Supplement: Supplementary file 1 — Supplementary Figures and Tables [file 41598_2017_5424_MOESM1_ESM.pdf]

Supplementary Information

Adaptive evolution of a hyperthermophilic archaeon pinpoints a formate transporter as a critical factor for the growth enhancement on formate

Hae Chang Jung[1,2,3], Seong Hyuk Lee[1,3], Sung-Mok Lee[1], Young Jun An[1], Jung-Hyun Lee[1,2], Hyun Sook Lee[1,2,*] & Sung Gyun Kang[1,2,*]

[1]Korea Institute of Ocean Science and Technology, Ansan, Republic of Korea. [2]Department of Marine Biotechnology, Korea University of Science and Technology, Daejeon, Republic of Korea. [3]These authors contributed equally to this work.

*Correspondence should be addressed to H.S.L (leeh522@kiost.ac.kr) or S.G.K. (sgkang@kiost.ac.kr).

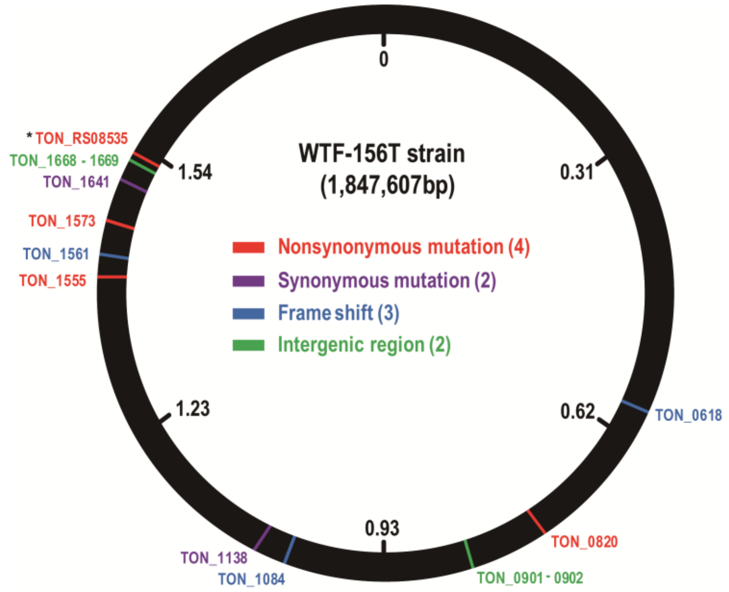

Supplementary Figure 1 | Schematic representation of mutations found in the genome of WTF-156T. The numbers inside and outside the circle represent genome position (Mb) and locus tag, respectively. Mutations are summarized in **Table 2**. The asterisk symbol (*) indicates newly assigned CDS applying new locus tag code : TON_RS00000.

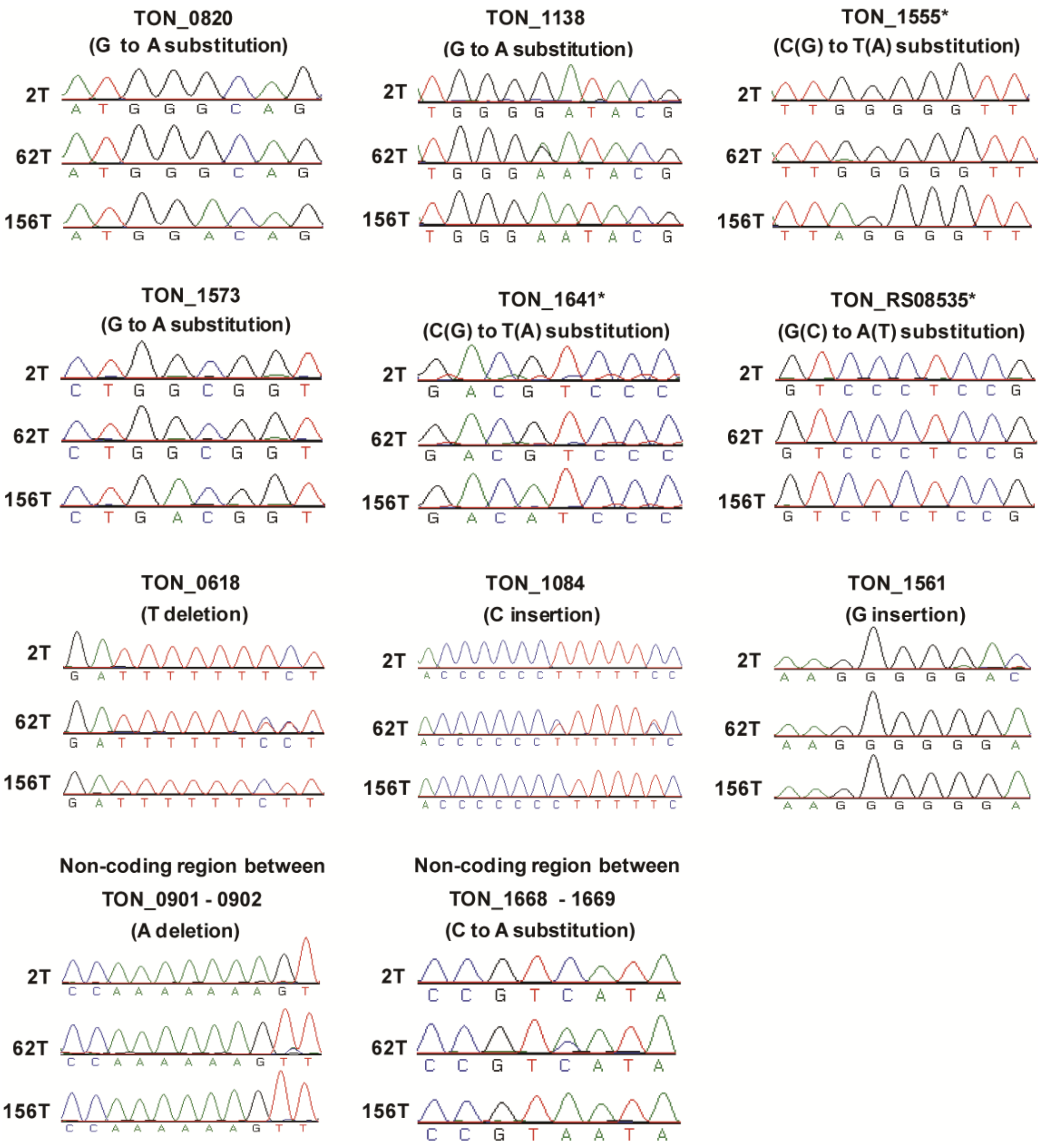

Supplementary Figure 2 | Confirmation of each mutation in the 2nd, 62nd and 156th transferred strains by PCR and Sanger sequencing. The asterisk symbol (*) indicates that complementary sequences were verified by Sanger sequencing.

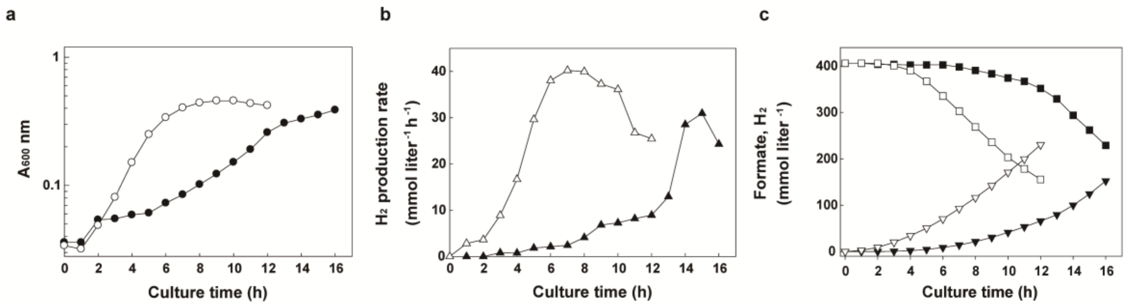

Supplementary Figure 3 | Effect of the mutation at TON_1573 (A52T) on formate utilization in a pH controlled bioreactor. Cell density (log $A_{600\ nm}$) (**a**) and H_2 production rate (**b**) in the parent (closed symbol) and mutant at TON_1573 (A52T) (open symbol) strains. (**c**), Changes of formate (square) and hydrogen concentrations (inverted triangle) in the parent (closed symbol) and TON_1573 (A52T) (open symbol) strains during the batch culture on 400 mM sodium formate. The pH was adjusted to 6.1-6.2 using 2 N HCl containing 3.5% NaCl as a pH-adjusting agent.

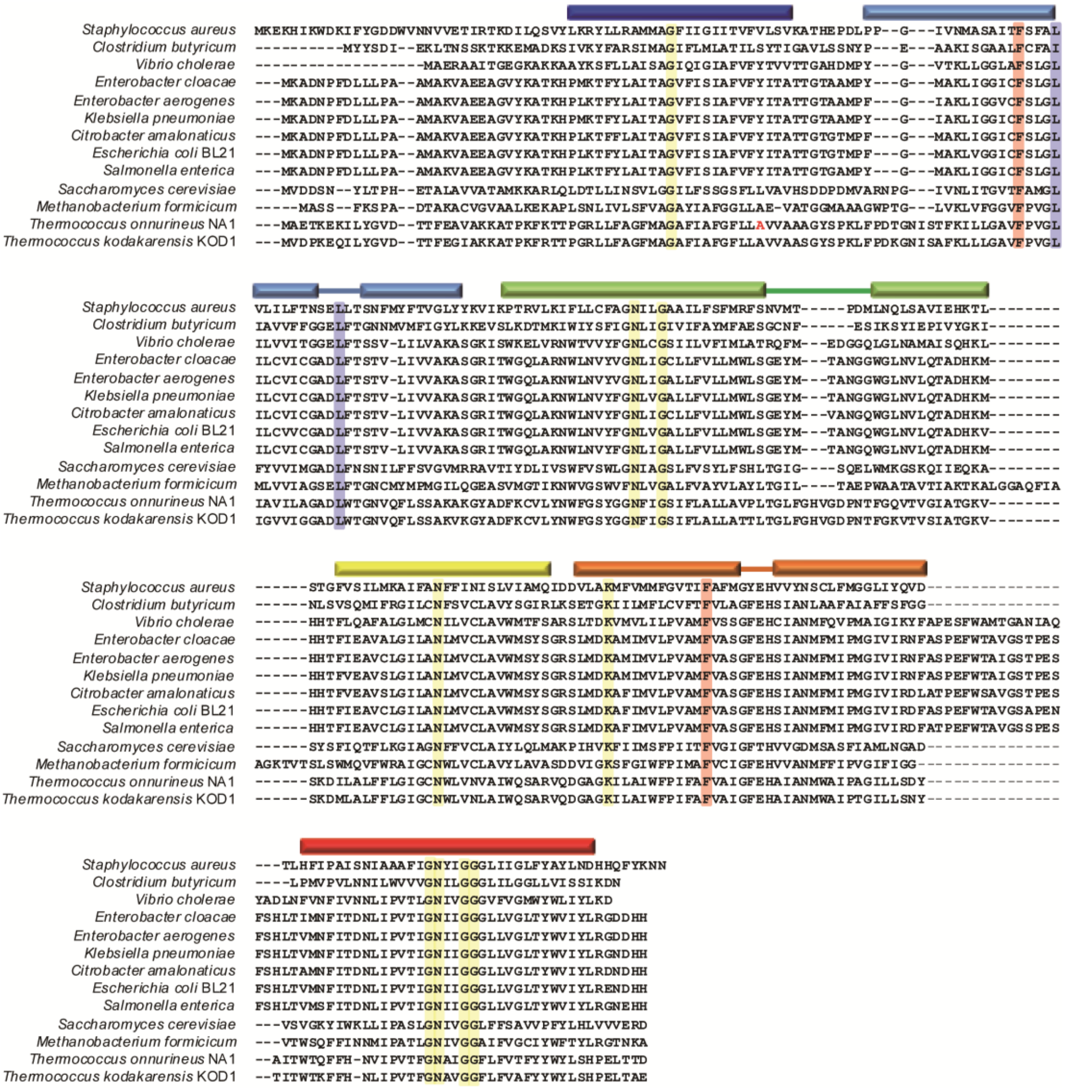

Supplementary Figure 4 | Sequence alignment of the formate transporter (TON_1573) with homologues from other formate-utilizing organisms. The candidate residues that influence the constriction sites are indicated in red and blue, and conserved amino acids are shaded yellow. The mutation residue (A52) of TON_1573 is highlighted in red. The GenBank accession numbers of sequences are as follows: formate nitrite transporter from *Staphylococcus aureus* (BAU33881); formate/nitrite transporter family protein from *Clostridium butyricum* (KHD14711); formate transporter from *Vibrio cholerae* (KKP19784); formate transporter from *Enterobacter cloacae* (AIV29111); formate transporter from *E. aerogenes* (KKY69429); formate transporter from *Klebsiella pneumoniae* (CTQ27319); formate transporter from *Citrobacter amalonaticus* (KOQ00258); FocA formate FNT transporter from *E. coli* BL21 (CAQ31432); formate transporter from *Salmonella enterica*

(KOP04599); hypothetical protein SCRG_04696 from *S. cerevisiae* (EDV09042); formate dehydrogenase from *Methanobacterium formicicum* (AAA73026); hypothetical formate transporter from *T. onnurineus* NA1 (ACJ17063); probable formate transporter from *T. kodakarensis* KOD1 (BAD86268).

Supplementary Table 1. The kinetic analysis of the parent and mutant at TON_1573 (A52T) strains

Kinetic parameters	Parent strain	TON_1573 (A52T) strain	Fold difference
μ_{max} (h^{-1})	0.3	0.62	2.05
r_{max} (mmol $liter^{-1}h^{-1}$)	31.7	40.2	1.27
Biomass productivity (g $liter^{-1}$ h^{-1})[a]	0.026	0.042	1.62
q_{max} (mmol g^{-1} h^{-1})	198.2	263.4	1.33
H_2 productivity (mmol $liter^{-1}$ h^{-1})[b]	9.5	19.2	2.02

Kinetic parameters were calculated using the data from graphs in Fig. 2. μ_{max} , maximum specific growth rate; r_{max}, maximum H_2 production rate; q_{max}, maximum specific H_2 production rate.

[a]Biomass productivity was determined by dividing the total yield by time difference from 11 to 13 h for the parent strain and from 4 to 6 h for mutant at TON_1573 (A52T) strains

[b]H_2 productivity was determined by dividing the total yield by time

Supplementary Table 2. The kinetic analysis of the recycling experiment

Kinetic parameters	Parent strain	Evolved strain*
μ_{max} (h^{-1})	0.3	0.43
r_{max} (mmol $liter^{-1} h^{-1}$)	31.7	85.8
Biomass productivity (g $liter^{-1}$ h^{-1})[a]	0.026	0.085
q_{max} (mmol g^{-1} h^{-1})	198.2	351.6
H_2 productivity (mmol $liter^{-1}$ h^{-1})[b]	9.5	70.9

Kinetic parameters were calculated using the data from graphs in Fig. 2. μ_{max} , maximum specific growth rate; r_{max}, maximum H_2 production rate; q_{max}, maximum specific H_2 production rate.

[a]Biomass productivity was determined by dividing the total yield by time difference from 11 to 13 h for the parent strain and from 2 to 4 h for repeated batch strains

[b]H_2 productivity was determined by dividing the total yield by time

*The data for kinetic analysis were adapted from Bae et al. (2015)

Supplementary Table 3. Mutations found in the strain at the recycling experiment

Locus_tag	Location	Mutational change	Product description
TON_0865	T356C	L119P	Pyridine nucleotide-disulfide oxidoreductase
TON_0916	G353A	G118D	ATPase C-terminus
TON_1513	C521T	A174V	Orotate phosphoribosyltransferase
TON_1573	C155A	A52E	Formate transporter
TON_1779	C422A	S141Y	ATPase
TON_0902	G426A	E142E	Biotin-protein ligase
TON_1532	C321T	Y107Y	Lipoate-protein ligase
TON_1872	C168T	P56 P	Putative vitamin B12 transport protein
TON_1513	C522T	A174A	Orotate phosphoribosyltransferase
TON_0536	617A deletion	Frame shift	Cytochrome-c3 hydrogenase subunit gamma

Supplementary Table 4. Primers used in this study

Primer	Oligonucleotide Sequence
**Construction of mutant**	
pUC118_0282del_HMG_fo_inverse_F	5'-gacctgcaggcatgcaagct-3'
pUC118_0282del_HMG_fo_inverse_R	5'-gactctagaggatccccggg-3'
TON_0820_SLIC_F	5’-ggatcctctagagtccaatactcgggaacctcaag-3’
TON_0820_SLIC_R	5’-gcatgcctgcaggtctctgggccgcgtacctctca-3’
TON_1084_SLIC_F	5’-ggatcctctagagtctcctgtcgcgtgaaggggct-3'
TON_1084_SLIC_R	5’-gcatgcctgcaggtcgctatccttcttccggtctt-3'
TON_1561_SLIC_F	5’-ggatcctctagagtcgatacaacgctggcactcat-3'
TON_1561_SLIC_R	5’-gcatgcctgcaggtccagcgaaataaagccctcag-3'
TON_1573-SLIC-F	5’-tttggtttcctcctgacggtggttgc-3'
TON_1573-SLIC-R	5’-ccgctgcaaccaccgtcaggaggaaa-3'
1573-point-mutation-F	5’-tttggtttcctcctgacggtggttgc-3'
1573-point-mutation-R	5’-ccgctgcaaccaccgtcaggaggaaa-3'
TON_1561_insertion(G)-F	5’-ggacatagtccttaagggggggacttc-3'
TON_1561_insertion(G)-R	5’-tcgaggaagtcccccccttaaggacta-3'
**Confirmation of constructs**	
TON_1573_point- confirm-R	5’-tgcaaccaccgt-3'
TON_0820_ point-confirm-R	5’-agaagacgctgc-3'
TON_1084_point-confirm-F	5’-cagaaccccccc-3'
TON_1561_point-confirm-F	5’-cttaagggggg-3'
**Confirmation of mutations in coding region**	
TON_0618-F	5’-cctcatttattccaaaacta-3'
TON_0618-R	5’-ctaaaataaaactttcagga-3'
TON_0820-F	5’-acagaggtgagagagatgcccgttac-3'
TON_0820-R	5’-gaaaaaagcaaaggattacttcctga-3'

TON_1084-F	5'-ataccctacgagcgctggta-3'
TON_1084-R	5'-tgcgttgaagttggccctaa-3'
TON_1138-F	5'-cctctacgggagggtgaaga-3'
TON_1138-R	5'-ccgaacctcgatcccggggg-3'
TON_1555-F	5'-gagatacccctccacagtca-3'
TON_1555-R	5'-tggtgatgttatcctataca-3'
TON_1561-F	5'-caagggaggagctccttgaa-3'
TON_1561-R	5'-tctgcgctctcgcaagcttt-3'
TON_1573-F	5'-atccttcgaacggtcatact-3'
TON_1573-R	5'-gtctccaacgtggccgaaga-3'
TON_1641-F	5'-acagcggtactcctcgcgct-3'
TON_1641-R	5'-ttcctagcgttaatcatata-3'
TON_RS08635-F	5'-tccttaaaattccagttccc-3'
TON_RS08635-R	5'-tagtttttgaacctcaagc-3'
**Confirmation of mutations in non-coding region**	
TON_0901-0902-intergenic region-F	5'-cgccaacccttccgagccgc-3'
TON_0901-0902-intergenic region-R	5'-ttctctgtcagaagtcttcc-3'
TON_1668-1669-intergenic region-F	5'-cccagcgcatagacatggtg-3'
TON_0901-0902-intergenic region-R	5'-cggctattgcagagccgccg-3'
